# How Does C-V2X Help Autonomous Driving to Avoid Accidents?

**DOI:** 10.3390/s22020686

**Published:** 2022-01-17

**Authors:** Lili Miao, Shang-Fu Chen, Yu-Ling Hsu, Kai-Lung Hua

**Affiliations:** 1 Department of Computer Science and Information Engineering, National Taiwan University of Science and Technology, Taipei 106, Taiwan; d10715005@mail.ntust.edu.tw (L.M.); chenshungfu@gmail.com (S.-F.C.); 2 Department of Industrial Management, National Taiwan University of Science and Technology, Taipei 106, Taiwan; yulinghsu@mail.ntust.edu.tw

**Keywords:** C-V2X, autonomous driving, PC5, RSU, OBU, VRUCW, AI

## Abstract

Accidents are continuously reported for autonomous driving vehicles including those with advanced sensors installed. Some of accidents are usually caused by bad weather, poor lighting conditions and non-line-of-sight obstacles. Cellular Vehicle-to-Everything (C-V2X) radio technology can significantly improve those weak spots for autonomous driving. This paper describes one of the C-V2X system solutions: Vulnerable Road User Collision Warning (VRUCW) for autonomous driving. The paper provides the system architecture, design logic, network topology, message flow, artificial intelligence (AI) and network security feature. As a reference it also includes a commercial project with its test results.

## 1. Introduction

Autonomous driving is growing to be part of the solution for better road safety, traffic efficiency, and environmental sustainability. Auto manufacturers and communication technology corporate are racing to develop the autonomous driving and related technologies, but reaching to the full driving automation is still a long way to go. There are total 6 levels for driving automation, ranging from 0 (fully manual) to 5 (fully autonomous) defined by Society of Automotive Engineer (SAE) [1] as listed in Table 1.

Many experts believe Level 4 and Level 5 won’t be commercialized until 2025 [2], however, the market demand of autonomous driving is tremendously increasing in these few years. Today we are seeing more and more partial driving automation vehicles with Level 2 or Level 3 on the roads. Due to the limited capability of these partially automated vehicles, they still require the driver to take control of the vehicle to deal with the urgent events. Unfortunately, if the driver dose not take any action and control the vehicle within enough time reserved, accidents could happen as reported in [3,4]. Therefore, the European Commission (EU) organized the Cooperative Intelligent Transport Systems (C-ITS), which allows road users and traffic managers to share information and use it to coordinate their actions. Nowadays many use cases [5] are deployed by C-ITS and some enhanced mechanisms were proposed. For example, having roadside equipment’s monitoring for vehicle movements and road conditions, detecting potential non-line-of-sight (NLOS) problems before a catastrophe happens.

Today, a serial of advanced sensors including IP Camera, Radar/LiDAR, and Advanced Driver Assistance Systems (ADAS) have been installed in autonomous driving vehicle. Sensors in the vehicle provide the basic functionalities of autonomous driving, it is crucial for safety and will not change in the near future, but these sensors are easily impacted by bad weather, poor lighting conditions or non-line-of-sight obstacles. Apparently, the automotive industry has recognized that connectivity is necessary to further increase the safety and comfort of Level 3 or Level 4 driving. Going beyond a certain level of driving autonomy will definitely require vehicles to be connected with each other or with network as mentioned in [6].

The cooperative C-V2X systems are known as vehicle-to-vehicle (V2V), vehicle-to-infrastructure (V2I), vehicle-to-pedestrian (V2P) or vehicle-to-network (V2N) communications. C-V2X can enable vehicle to communicate with other vehicle, infrastructure, pedestrian and network to improve road safety and road efficiency, as introduced in [7]. Our work is inspired by a number of prior studies on the applications of C-V2X. A brief summary of relatedworks is shown in Table 2.

In [8,9], the authors shared function and performance tests in V2V/V2I whose aim was to review how the automated driving benefits on PC5/Uu connected in LTE-V2X network to improve road safety and traffic efficiency. Moreover, V2X platform or MEC was used to send messages to RSU, and then RSU could broadcast them to vehicles through the PC5 interface. RSU also might send messages to network through the Uu interface. In [10], author solved problems of Uu interface based C-V2X and verified the performance of the solution in both LTE-V2X and NR-V2X networks. Then, ref. [11] suggested some pricing and investment strategies which may be helpful for a successful deployment of V2N. Refs. [12,13] focused on vulnerable road users (VRU) scenario, collision prediction algorithms were proposed based on pedestrian-to-vehicle (P2V) or vehicle-to-pedestrian (V2P) communication. In terms of network topology, they involved remote cloud-based cellular architecture and MEC infrastructure to decrease E2E communication latency. Refs. [14,15,16] concluded that Artificial Intelligence (AI) technology could make the collision avoidance system adapt to the environment and facilitate fast and accurate decisions. Edge analytics architecture was proposed to reduce AI computing time.

With the popularity of C-V2X technology in recent years, on the one hand, automobile manufacturers would like to bind C-V2X function to the future electric vehicles in order to strengthen safety. On the other hand, current driver does not need to wait until these C-V2X products are initially embedded in new manufactured vehicle models after few years. Instead, as the global automotive aftermarket, external C-V2X On-Board Unit (OBU) equipment can be installed flexibly in a traditional vehicle or autonomous vehicle that have left the factory already, to pioneerly experience C-V2X services based on existed Road-Side Unit (RSU) or telecommunication cellular network as mentioned in [17,18].

## 2. Solution for C-V2X VRUCW

Vulnerable Road User Collision Warning (VRUCW) is one of the most important C-V2X system solutions. It can help vehicle to avoid accidents. In this section, system integration, system architecture, network topology and system message flow will be introduced in details.

### 2.1. Autonomous Driving System Integration with C-V2X

**Figure 1:**
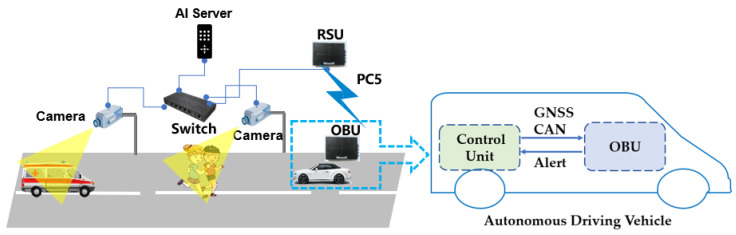
Autonomous Driving Integration with C-V2X OBU.

As shown in Figure 1, we designed the system integration solution between C-V2X OBU and autonomous driving control unit in real life [19]. Because the specification of OBU is a standard as a vehicle equipment including operating system, power supply, operational temperature and radio frequency band, this integration can adapt to all kinds of vehicles as long as they are in accordance with the international standards.

Firstly, RSU as a roadside equipment receives information from outside sources, such as AI servers, 4G/5G telecommunication network. Then, it broadcasts this information by PC5 interface using a unified message format in its coverage field, the maximum reachable radius can be one kilometer if there are not obstacles such as buildings or trees. Secondly, OBU is connected with control unit Industrial PC (IPC) of the autonomous driving system via Transmission Control Protocol (TCP)/Internet Protocol (IP) protocol. The OBU with 12V power is provided by vehicle, and antennas of OBU should be installed outside of vehicle for better messages receiving. After a successful 3-way TCP/IP handshake connection between OBU and control unit of autonomous vehicle, OBU can receive Global Navigation System Satellite (GNSS) and Controller Area Network (CAN) messages from the vehicle’s control unit. Thirdly, combining with vulnerable road user information from RSU, OBU can instantly judge the status of road safety and notify autonomous driving control unit.

### 2.2. C-V2X VRUCW Deployment

#### 2.2.1. System Architecture

**Figure 2:**
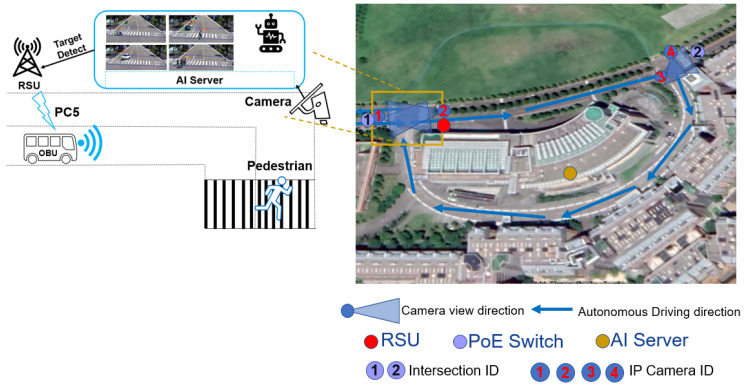
C-V2X VRUCW System Architecture.

Based on the above system architecture in Figure 2, we select one circular route to deploy and verify our solution. Normally, autonomous driving vehicle cannot know any NLOS object by its own IP camera from a long distance. For instance, there is one person is running at 500 meters beyond with a 90-degree corner of the street. To meet high cost-performance demand, one RSU is installed at site as shown by red color dot, it is sufficient to cover both intersection 1 and 2. The orange color dot is AI server position. For monitoring pedestrians in different road directions, we build up two IP Cameras at the left intersection, and two IP Cameras at the right intersection. Two Power over Ethernet (PoE) switches are used as a whole network backbone connection.

#### 2.2.2. Network Topology

**Figure 3:**
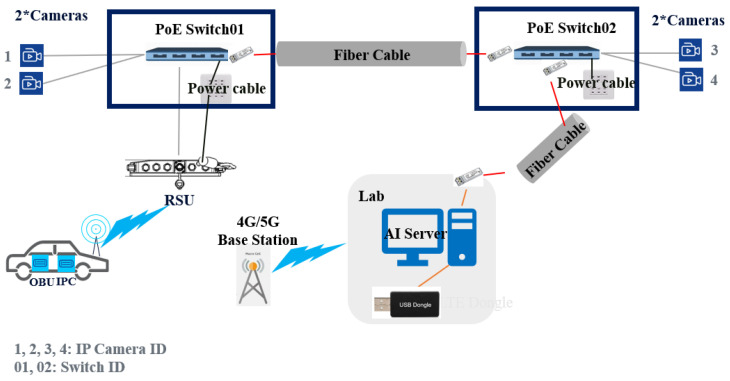
C-V2X VRUCW Network Topology.

**Figure 4:**
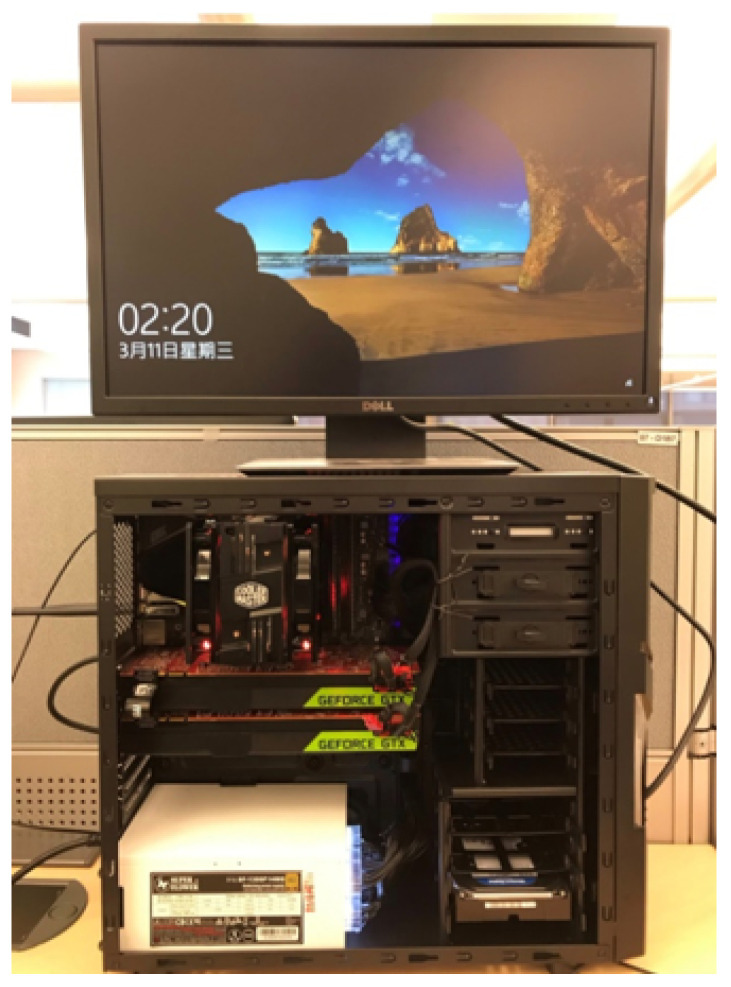
AI Server HW/OS.

According to live environment, network topology is designed in Figure 3. In order to avoid accidents in blind spot, first, RSU with maximum 1-kilometer reachable radius coverage is connected with PoE switch. Next, IP Cameras 1 & 2 access network by PoE switch 1, and IP Cameras 3 & 4 access network by PoE switch 2. Four IP Cameras make video streaming and transfer all instant data to the central AI server which is placed in the middle of the circular route. The AI server as showed in Figure 4 is responsible for artificial intelligent workload in our solution. The hardware specification of the AI server is listed in Table 3.

We verified the computation time of the AI program in our lab. Detection latency is the duration between decoding an image data and sending the recognition result from the socket of program, final result is summarized in Table 4. Certainly, the ∼166 ms latency is too high to meet real-time requirement of advanced C-V2X services, but it is acceptable in our solution because we intend to offer the basic road safety strategy and focus on functional testing as a proof of concept project. We need a tradeoff between real-time constraints and the investment project. Furthermore, we plan to deploy AI workload in the roadside smart sensor level in order to overcome the intelligent transport system (ITS) latency and reliability challenges.

**Figure 5:**
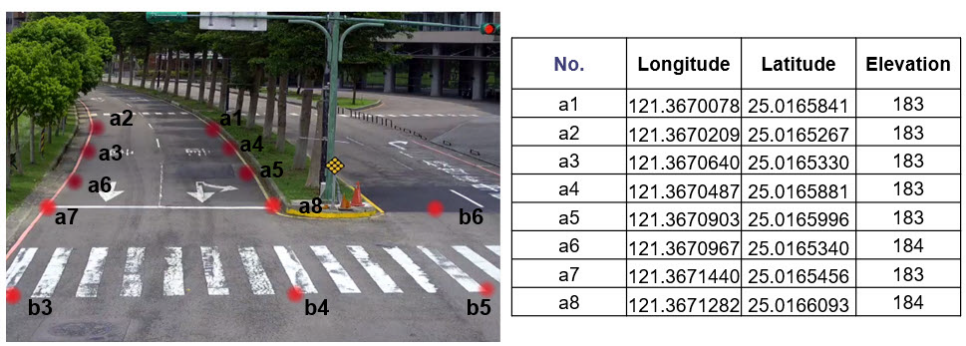
Position Information under Camera Coverage.

Under all cameras coverage, a geographic profile with latitude, longitude and elevation information is predefined in AI server, which is measured humanly by handheld Global Positioning System (GPS) tool, an example for one of four cameras is shown in Figure 5.

#### 2.2.3. System Message Flow

**Figure 6:**
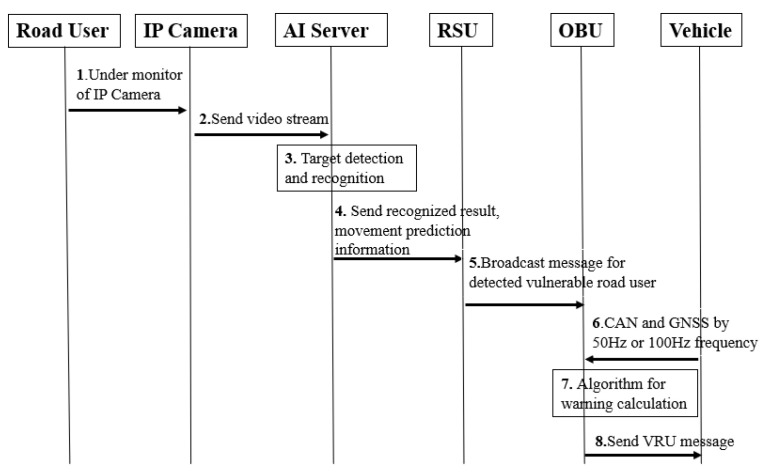
System Message Flow for C-V2X VRUCW.

According to Figure 6, IP camera sends video stream to AI Server. The image size 2592 × 1944 and image extraction speed 10 frames per second are used in our solution. The AI server equipped with the target classification algorithm is utilized to determine the pedestrian’s category results, which include person, car, motorcycle, person with umbrella, baby carriage and person at night. If pedestrian passes this area under camera coverage, AI server will detect this object and transfer object recognition, position information and movement prediction analysis results to RSU. RSU encapsulates these messages into broadcast packages and broadcasts these information to all the OBUs within the coverage area of RSU. Meanwhile, autonomous driving vehicle’s control unit sends GNSS and CAN messages to OBU by 50 Hz or 100 Hz frequency. OBU is responsible for combining all the information to determine if a collision is approaching. We use below Algorithm 1 for collision warning trigger threshold after detecting an object. *S* is the distance between host vehicle and pedestrian. *V* is the speed of host vehicle. When all the trigger points are met for alert threshold, a VRU warning message will be sent out from OBU to autonomous vehicle control unit. Autonomous vehicle own control policy for slowing down or stopping is out scope of our solution.

**Algorithm 1** Threshold for VRUCW Trigger in OBU
1:**if***S* > 50 m **then**2:    the target pedestrian is keeping a safe distance with the host vehicle, no collision warning is sent.3:**else if***V* < 10 km/h **then**4:    the host vehicle speed is not fast so that pedestrian can walk away from danger situation, no collision warning is sent.5:**else if**S⩽ 50 m and V⩾ 10 km/h **then**6:    the target pedestrian is keeping a danger situation with the host vehicle, collision warning is sent.7:
**end if**



Threshold parameter values for *S* and *V* can be changed flexibly according to requirement.

## 3. Key Technology for C-V2X VRUCW

### 3.1. C-V2X

In our VRUCW solution, Qualcomm 9150 commercial chipset [20] is installed in both RSU and OBU to provide C-V2X PC5 interface function. According to free space path loss model in [21], the dBm value of received signal strength (RSS) can be given in Equation (1)

(1) $$R=10lg(\frac{16\pi^{2}}{\lambda^{2}}d^{\alpha })$$

where *R* is received signal strength; *d* is the distance between sender and receiver; path loss exponent of α = 2.2 is assumed; λ is the wavelength, can be derived from Equation (2):

(2) $$\lambda =\frac{c}{f}$$

*c* is light speed; *f* is the frequency of radio wave, we use *f* = 5.850–5.925 GHz, so when *R*⩾−125 dBm, *d*⩽ 1000 m as concluded in [22], after evaluation, one RSU is sufficient to cover both intersection 1 and 2 in our solution. Taiwan National Communications Commission (NCC) is planning to reserve 5.850–5.925 GHz for Intelligent Transport Systems (ITS) development when writing this paper. 5G Automotive Association (5GAA) [23] has called on national and regional regulators to make spectrum 5.855–5.925 GHz band available for global harmonized use. The U.S. Federal Communications Commission (FCC) [24] selected the frequency range 5.850–5.925 GHz to improve automotive safety. The European Telecommunications Standards Institute (ETSI) harmonizes the 5.875–5.905 GHz frequency band for ITS applications. While China are using 5.905–5.925 GHz for ITS trials and considers C-V2X the only option. Though most countries and regions make the spectrum technology neutral, C-V2X is favored by almost all major telecommunication operators, auto makers, and technology vendors. ITS Spectrum for V2X in global is summarized in Table 5.

### 3.2. AI

**Figure 7:**
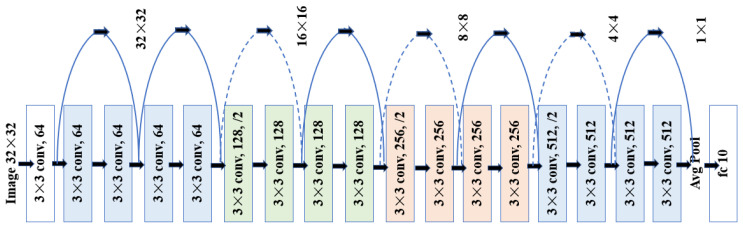
ResNet-18 Network [25].

Artificial Intelligence (AI) detection, object recognition and movement prediction for collision warning are used in our solution. In AI server, we implement SSD and ResNet-18 [25] network framework. SSD stands for Single Shot Detection, it means that AI server only needs to take one single shot to detect multiple objects within the image. Compared with the two-stage image detection, the inference and error rate are greatly reduced. The deep residual network (ResNet) is one of the most commonly convolution neural networks (CNNs) for the image feature extraction. ResNet-18 consists of 17 convolution layers and a fully connected layer as shown in Figure 7. This neutral network reduces the amount of calculation by using a 3 × 3 small convolution kernel, so that the time consumption of handling process can meet the low latency required by C-V2X in 3GPP Rel-14 [7].

A confusion matrix [26] in Table 6 is a technique for summarizing the performance of a classification algorithm. Most widely-used metric is Equation (3):

(3) $$Accuracy=\frac{TP+TN}{TP+TN+FP+FN}$$

But classification accuracy as an evaluation measure is not well-suited for imbalanced class. Detecting the rare class is usually more interesting (e.g., person with umbrella, baby carriage, etc). So we have alternative measures for classification performance evaluation in Equations (4)–(6).

(4) $$Precision=\frac{TP}{TP+FP}$$

(5) $$Recall=\frac{TP}{TP+FN}$$

(6) $$F1=\frac{2\times Precision\times Recall}{Precision+Recall}=\frac{2\times TP}{2\times TP+FP+FN}$$

According to Equations (4)–(6), two features person and vehicle are classified in testing set, experimental result is showed in Table 7.

**Figure 8:**
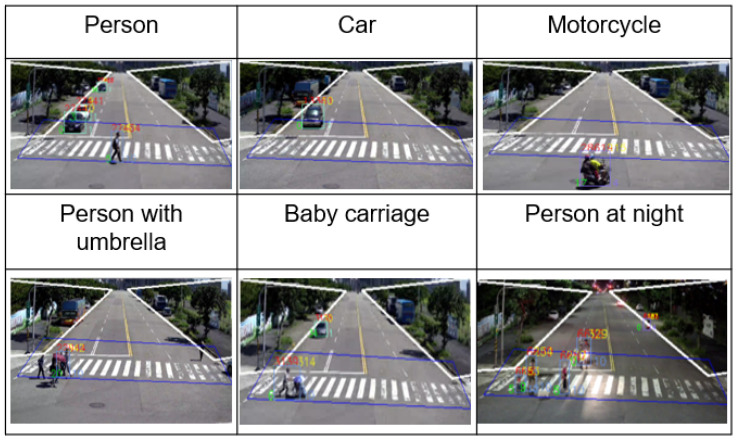
Vulnerable Road User Category.

From Figure 8, the vulnerable road user as a detection result can be a single person, a baby carriage, car, or motorcycle, etc. No matter it is daytime or nighttime, these vulnerable road users can be protected by our C-V2X VRUCW solution. If any new target appears that does not belong to our existed AI database, but there is a possibility of collision between new target and autonomous vehicle, considering the risk of road accidents, new target will be treated as a person or a car in terms of default setting in our solution.

### 3.3. Security Support

Even though the essential security and privacy aspects of C-V2X specified by 3GPP are introduced in [27], there is no security details definition in V2X application layer in Rel-16 specification [28], i.e., confidentiality and integrity protection for group communication. Therefore, prior to marketization of C-V2X technology services deployment, government must ensure the security and privacy of C-V2X Sidelink transmission in a reliable and trust level, and protect C-V2X Sidelink transmission from hacker’s attacking, e.g., Jamming, Eavesdropping and others as introduced in [29,30,31]. In this paper, we neither address complicated 5G core network security policy in C-V2X V2N mode where authentication is stated as the procedure of verifying the user before providing access to the system and only authorized user can have access rights to the network, nor involve third party Certificate Authority (CA) which requires government by using unified regulations to drive authority agency, nor provide hardware security model (HSM) which is easy to trigger higher cost investment based on available hardware capability in current market. Instead, our approach does not require any additional infrastructure, we design a symmetric software encryption function for C-V2X Sidelink transmission which is suitable for early C-V2X technology market. An original system architecture of security support for C-V2X VRUCW service is firstly proposed.

**Figure 9:**
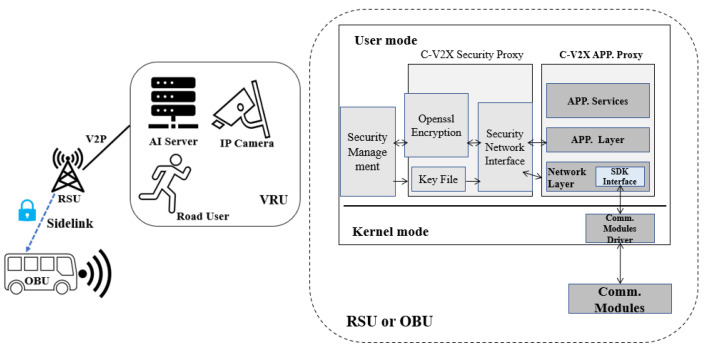
Security Support for C-V2X RSU/OBU.

In Figure 9, the entire design for security support of C-V2X Sidelink transmission is consists of two resident modes (User mode and Kernel mode) and a key profile management tool. The security process is proceeded between the network layer and application layer of C-V2X RSU/OBU program simultaneously. C-V2X Security Proxy and C-V2X APP Proxy use Inter-Process Communication method. When C-V2X APP. proxy detects that security function is turn on, the upcoming packages will be sent to C-V2X security proxy by security network interface for software symmetric encryption algorithms process. Advanced Encryption Standard-Electronic Codebook (AES-ECB) mode as our selection is enough to provide a secure cipher procedure for encrypting and decrypting some sensitive string values in C-V2X Sidelink transmission. Key file is managed by key profile generation tool. Symmetric encryption key has been copied into both RSU and OBU devices manually in advance. Symmetric means ciphers use the same key for encrypting and decrypting, so the sender and the receiver must both know and use the same secret key.

**Figure 10:**
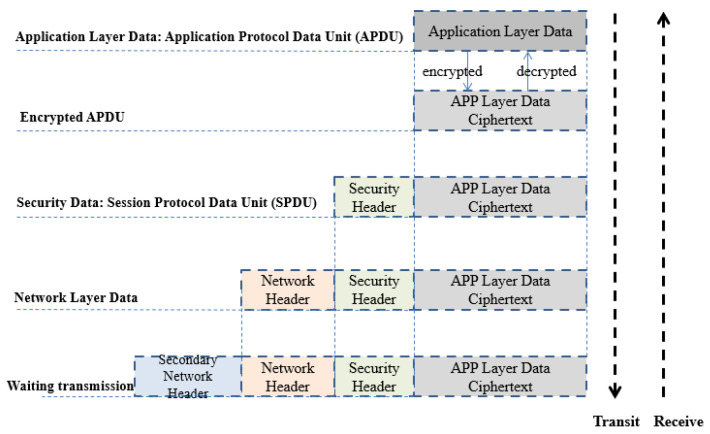
Security message header definition.

**Figure 11:**
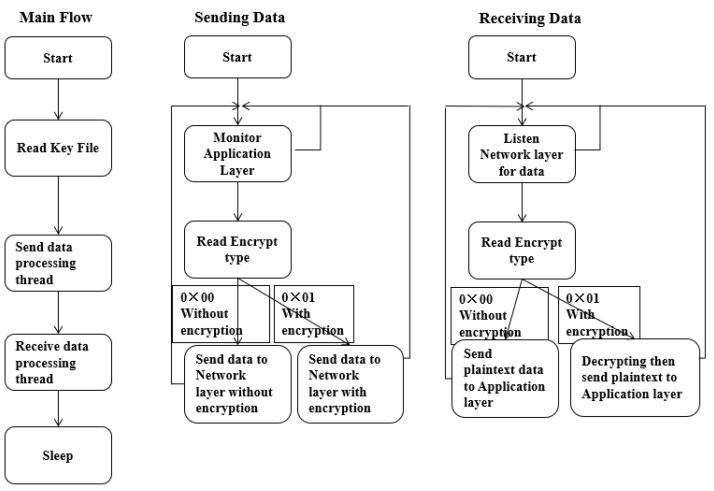
Security message flow definition.

According to message header definition in Figure 10, security packet messages are encapsulated and de-encapsulated as designed. In Figure 11, before wireless device communication with security support, we first generate key profile by using our key file management tool, then input same key files into C-V2X RSU and OBU in both Sending/Receiving thread, active or inactive security function in both devices keeps same. During RSU sending data, its C-V2X security proxy is responsible for monitoring and reading the status of C-V2X application proxy, if encrypt type “0 × 01” is gotten, data will be sent to security proxy for encryption progress. Another communication side, OBU is listening on receiving data, when encrypt type “0 × 01” encryption is detected, it will proceed decryption procedure then send plain-text to application layer of device for executing C-V2X services such as our VRUCW solution for example. Without same key profile or encrypt type, RSU and OBU cannot talk each other so as to achieve a guarantee of C-V2X Sidelink transmission security in the field experiment.

We admit that AES-ECB mode in our solution has some known weakness, which there is still possibility that an attacker breaks into our database and steals our data after their many planned entries. But as proof of concept as we emphasize, our target is to address a workable security support for C-V2X during the early stage deployments in some countries. After C-V2X technology is evolved to mature stage, 5G Core Network function unit Authentication Server Function (AUSF), the third-party CA, or HSM would be preferred by market.

## 4. Testing and Demonstration

A commercial C-V2X project [32] is deployed in Chang Gung Health and Culture Village, Taoyuan City, Taiwan. In order to cooperate with autonomous driving vehicle, our solution is selected for C-V2X VRUCW application to provide additional protection for the aged residents as a technological transformation strategy in the village.

### 4.1. Function Test

**Figure 12:**
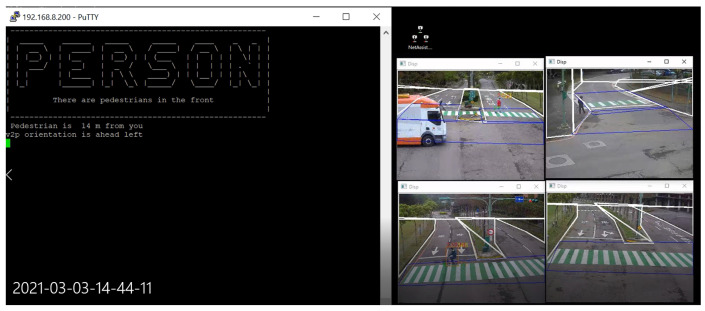
VRUCW Function Test.

By means of real time streaming protocol (RTSP) media stream technology, as specified in [33], the view screens of four IP cameras are put on one display desktop of a computer together. This means that whether road users can be detected or recognized is intuitively observed. Meanwhile, we build up a putty tool [34] to access linux system of OBU device; then, we can monitor both road users and status of OBU alarm trigger at same time. When one person is passing the road, as seen in camera screen of the bottom-left corner, according to message flow in Figure 6 and Algorithm 1, once the OBU of autonomous driving vehicle detects a potential collision possibility, a warning message “PERSON” as designed will pop up in OBU putty tool as showed in Figure 12. Simultaneously, OBU sends this warning message to autonomous driving control system for slowing down or stopping. Finally, with our proposed solution, we have successfully launched the C-V2X VRUCW function in the field. It proves that our solution is workable and can help autonomous driving to avoid accidents, especially in NLOS situation.

### 4.2. PC5 Latency Test

**Figure 13:**
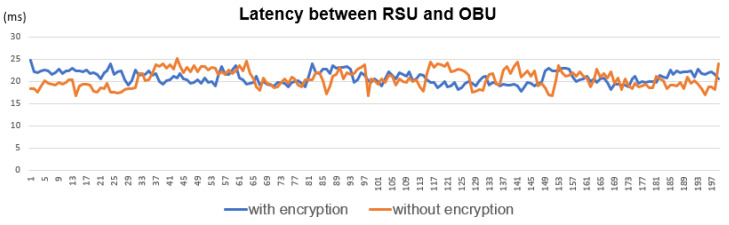
PC5 Latency Test.

Because VRUCW is time-sensitive service, we are worried that low latency performance can’t be satisfied due to adding extra software security function. To eliminate this concern, we implemented C-V2X PC5 Sidelink transmission latency testing before and after security function activation in Figure 13.

From the test result in Table 8, it proves that C-V2X PC5 transmission performance is not impacted after adding additional security function.

## 5. Discussion

For our solution, we need consider these limitations: (1) Camera coverage area is limited, we need more cameras for full coverage, it depends on investment cost; (2) Manually measuring geographic information for latitude, longitude and elevation and configuring in AI Server, it is easy to cause deviation and impact message trigger accuracy. In future, we prefer to use multiple probe tools such as smart camera and LiDAR sensor fusion solution for automatic target position detection; (3) PC5 interface latency is around 20 ms, and plus fiber-optic/cable transmission delay and AI computation time, end-to-end latency is within 200 ms. In general, our solution is effective for autonomous driving to avoid NLOS accidents, which can meet basic road safety requirement as introduced in 3GPP Rel-14 [7]. In future, in order to deploy more advanced services such as remote driving and platooning as introduced in 3GPP Rel-15 [35], we will try AI workload in roadside smart sensor level to overcome the high latency issue. When 5G New Radio (NR) network is ready in the field, we will implement C-V2X applications based on URLLC (Ultra-Reliable Low-Latency Communication) of 5G NR-V2X as described in 3GPP Rel-16 [36].

## 6. Conclusions

Based on our novel C-V2X system solution with autonomous driving, the original designs for system architecture, network topology and service message flows are showed. After testing and demonstration in open filed, it proves that our solution for VRUCW application is workable and can increase road safety for autonomous driving. Meanwhile we provide the security support with software symmetric encryption in our solution, it is helpful for C-V2X deployment in early stage of some countries as proof of concept, which fills the research gap of security support in 3GPP Rel-16 V2X specification that there is no security details definition in V2X application layer. In addition, as the key research in the future work, we will try software asymmetric encryption solution to make C-V2X Sidelink transmission more securely.

## Figures and Tables

**Table 1:** Autonomous Driving Level [1].

Level	Name	Fallback When Automation Fails
0	No Driving Automation	Driver
1	Driver Assistance	Driver
2	Partial Driving Automation	Driver
3	Conditional Driving Automation	Driver
4	High Driving Automation	Automated system
5	Full Driving Automation	Automated system

**Table 2:** Literature Summary.

Use Case	Source	Research Subject
V2V and V2I	[8,9]	PC5 or Uu interface, V2X Server/MEC
V2N	[10,11]	NodeB-based interface, V2X Server/MEC
V2P	[12,13]	Uu interface, V2X Server or 5G/MEC
AI-based solution	[14,15,16]	AI algorithms, edge analytics architecture

**Table 3:** AI Server Hardware Specification.

Hardware	Specification
CPU	I7-8700K
GPU	2 × GTX1080Ti
RAM	256 G
Hardisk	1 TB
OS	Windows 10

**Table 4:** AI Program Latency.

Item	Step	Latency (ms)
	Preprocess	1.3697
	Detect	165.0450
Detect Program	Convert result	0.0071
	Save result	0.0403
	Total	**166.4621**

**Table 5:** ITS Spectrum [23].

Country	Spectrum (MHz)	Allocated Bandwidth (MHz)
Australia	5855–5925	70
China	5905–5925 (trials)	20
Europe	5875–5905	30
Japan	755.5–764.5 and 5770–5850	9 and 80
Korea	5855–5925	70
Singapore	5875–5925	50
U.S.	5850–5925	75

**Table 6:** 2-class Confusion Matrix [26].

	Predicted Class	Positive	Negative
Actual Class			
**Positive**		True Positive **(*****TP*****)**	False Negative **(*****FN*****)**
**Negative**		False Positive **(*****FP*****)**	True Negative **(*****TN*****)**

**Table 7:** Classification Performance.

Category	Precision	Recall	F1
Person	98.61%	91.79%	95.08%
Vehicle	95.73%	99.70%	97.68%
Average	97.84%	95.71%	96.71%

**Table 8:** Latency Test Result.

PC5 Interface	With Encryption	Without Encryption
Test times	200	200
Average latency	20.83 ms	20.53 ms

## Data Availability

Data available on request from the authors.

## Abbreviations

The following abbreviations are used in this manuscript:

AI	Artificial Intelligence
ADAS	Advanced Driver Assistance System
AUSF	Authentication Server Function
AES-ECB	Advanced Encryption Standard- Electronic Codebook
C-V2X	Cellular Vehicle-to-Everything
C-ITS	Cooperative Intelligent Transport Systems
CAN	Controller Area Network
CNN	Convolution neural network
CA	Certificate Authority
CPU	Central Processing Unit
EU	European Commission
ETSI	European Telecommunications Standards Institute
FCC	Federal Communications Commission
GNSS	Global Navigation System Satellite
GPS	Global Positioning System
GPU	Graphics Processing Unit
HSM	Hardware Security Model
ITS	Intelligent Transport Systems
IP	Internet Protocol
IPC	Industrial Personal Computer
LiDAR	Light Detection and Ranging
LOS	Line-of-Sight
LTE	Long-Term Evolution
MEC	Multiaccess Edge Computing
NLOS	Non-Line-of-Sight
NCC	National Communications Commission
NR	New Radio
OBU	On-Board Unit
PoE	Power over Ethernet
VRUCW	Vulnerable Road User Collision Warning
RAM	Random Access Memory
ResNet	Residual Network
RSU	Road-Side Unit
RSS	Received Signal Strength
RSTP	Real Time Streaming Protocol
SAE	Society of Automotive Engineer
SSD	Single Shot Detection
TCP	Transmission Control Protocol
3GPP	The 3rd Generation Partnership Project
UE	User Equipment
URLLC	Ultra-Reliable Low-Latency Communication
V2V	Vehicle-to-Vehicle
V2I	Vehicle-to-Infrastructure
V2P	Vehicle-to-Pedestrian
V2N	Vehicle-to-Network
